# Health-related quality of life and its predictors among hypertensive patients on follow up at public hospitals in Addis Ababa, Ethiopia: application of Tobit regression model

**DOI:** 10.1186/s13104-024-06787-8

**Published:** 2024-05-03

**Authors:** Yordanos Megerssa, Guta Kune, Mamo Nigatu

**Affiliations:** 1 Medical and Sales Representative at Beker General Business PLC, Addis Ababa, Ethiopia; 2 Department of Epidemiology, College of Public Health, Jimma University, Jimma, Ethiopia

**Keywords:** Health-related quality of life, Hypertension, Public hosp itals, Addis Ababa

## Abstract

**Background:**

Health-related quality of life and its associated factors among hypertensive patients living in Ethiopia are not well studied. Therefore, this study aims to assess the level of health-related quality of life and its associated factors in hypertensive patients on follow-up in Public Hospitals in Addis Ababa, Ethiopia.

**Methods:**

A facility-based cross-sectional study was conducted among 339 hypertensive patients on follow-up at Yekatit 12 &Zewditu Hospitals. Data were collected through face-to-face interviews using Euro Quality of Life Groups 5 Dimensions 5 Levels (EQ-5D-5L) in combination with Euro Quality of Life Groups Visual Analog Scale (EQ-VAS). A multivariable Tobit regression model was employed to assess the association between EQ-5D-5L index, EQ-VAS, and potential predicting factors.

**Results:**

The median index value and EQ-VAS Scales score was 0.86 (IQR = 0.74, 0.94) and 69 (IQR = 55, 80) respectively.

The proportion of participants reporting anxiety/depression and pain/discomfort problems was highest, while the fewest patients reported problems in the self-care dimension. Older, rural residents, low income, higher stages of hypertension, increased use of antihypertensive medications, and patients with an increased hospitalization rate scored lower on health-related quality of life than others.

**Conclusion:**

Health-related quality of life among hypertensive patients attending public health hospitals in Addis Ababa is unacceptably poor. Emphasis should be given to patients with higher stages of hypertension, increased use of antihypertensive medications, and an increased hospitalization rate giving due focus to older, rural residents, and low-income patients to promote their health-related quality of life.

## Introduction

Non-communicable diseases (NCDs) are a major public health concern that continues to cause significant death and morbidity around the world [1]. Non-communicable diseases (NCDs) are the world's leading causes of death and disability, making them humanity’s most pressing health concern in the twenty-first century [2]. Hypertension is one of the key public health issues due to its high prevalence and related dangers such as cardiovascular and kidney illnesses, which can range from myocardial infarction to stroke and renal failure [3].

Hypertension is a primary cause of morbidity and mortality, and its incidence is on the rise, particularly in developing countries [4]. The major goal of hypertension treatment is to reduce long-term cardiovascular risks, but health-related quality of life (HRQoL) in hypertensive patients has recently received increased attention to improve daily functioning, minimize physical and psychological pain, and allow full participation in social life [5]. Hypertension can affect a patient's physical health, psychological well-being, level of independence, and familial and social interactions, resulting in a decrease in HRQoL [6]. Compared to healthy people, hypertensive patients have lower HRQoL, which is affected by blood pressure, organ damage, comorbidities, and treatment [5].

Health-related quality of life is a major concern of patients, healthcare professionals and policy makers and has received much attention in recent years [4]. HRQoL exams provide important and subjective information about a person’s mental and physical health in everyday life [7]. In terms of physical and mental health outcomes, HRQoL is an important indicator for NCDs [8]. Health-related quality of life scales capture the patient's perspective, which is critical in providing patient-centered and collaborative care that is valuable to patients. Measuring HRQoL helps doctors become more aware of their patients' issues and, more significantly, enhances patient-clinician dialogue [9].

Even though many nations are interested and concerned with the impact of hypertension on HRQoL [4, 5, 7, 10, 11] little is known about HRQoL among hypertensive patients living in Ethiopian and its correlations with socio-demographic features and clinical conditions. Only two investigations have been conducted so far [11, 12]. Therefore, in order to gain a better understanding, this study is taken up to assess the level of HRQoL and related factors in hypertensive patients at follow-up in public hospitals in Addis Ababa, Ethiopia.

## Methods

### Study design, setting, participants, and period

A facility-based cross-sectional study was conducted among hypertensive patients on follow-up visits at two randomly selected public hospitals in Addis Ababa; Yekatit 12 and Zewditu Memorial Hospital,Ethiopia. Critically ill hypertensive patients p who were not able to respond to the questionnaire were excluded from the study.

### Sample size determination and sampling techniques

The sample size was determined using a single population mean formula by considering the following assumptions: 19 standard deviation of health-related quality of life (HRQoL) scores among hypertensive patients from the previous study [11], 95% confidence interval, 3% margin of error, 2 design of effect, and 10% non-response rate. Hence, the study was conducted among 339 finally calculated hypertensive patients. Multistage random sampling was used to recruit the study participants.

### Data collection tools, procedures, and quality assurance

The questionnaire had three parts, Euro Quality of Life Group’s 5- Dimensions 5 Levels (EQ-5D-5L) tool, socio-demographic characteristics, and clinical factors. The EQ-5D-5L has been translated to Amharic, using the standardized approach recommended by the European Quality of Life (EuroQol) group [13]. The Amharic version of EQ-5D-5L was used to collect data which was acquired from the EuroQol research foundation upon request by the principal investigator.

#### EQ-5D-5L

The generic EQ-5D-5L questionnaire which consists of five dimensions, further divided into five levels of severity. The five dimensions: (i) mobility, (ii) self- care, (iii) usual activities, (iv) pain/discomfort, and (v) anxiety/depression of EQ-5D are self-reported by patients. Each dimension has a five-level scale (no problems, slight, moderate, severe, and extreme) scored from 1 to 5 [14].

#### EQ-5D index

From the five dimensions of EQ-5D, a single index value, called EQ-5D index was calculated by using the population preference scores of Ethiopia as the reference. The value is derived from the Ethiopian general population survey to derive the EQ-5D index (16). The EQ-5D index ranges from 0 to 1, where 0 indicates severely ill, and 1 indicates a perfect health. Perfect health is represented by no problems on all five dimensions (11111) and is assigned an index value of 1. Likewise, very severe health states, corresponding to severe problems on all of the five dimensions (55555), received 0 values [13].

#### Euro quality of life group’s visual analytic scale (EQ-VAS)

Euro Quality of Life Group’s Visual Analog Scale (EQ-VAS) is a vertically calibrated scale that allows participants to rate their overall health on a scale ranging from 0 to 100; where 0 and 100 signify the worst and the best imaginable health state, respectively [13]. During data collection, each participant was given a pen and asked to indicate the point on EQ-VAS that they felt best described their overall health on that day.Before commencing actual data collection, a pretest was done on 5% (17) of hypertensive patients attending Menelik II referral hospital. Findings and experiences from the pre-test were utilized in modifying the questionnaire. Data were collected through face-to-face interview method by trained two diploma nurses and one BSc nurse was recruited for supervision purpose. Patients were interviewed after they got the services.All the collected data were checked by the supervisor daily for completeness and finally, the principal investigator monitored the overall quality of data collection.

### Data analysis

The collected data were checked for completeness and consistency and coded manually. Data were entered into Epi-Data v.3.1, Statistical analysis was performed using SPSS v. 25, and STATA v. 14 was used for further analysis.Descriptive statistics were used to present demographic and clinical characteristics of the study participants. intergroup differences in EQ-5D-5L index and EQ-VAS scores were assessed for statistical significance using either Mann–Whitney or Kruskal–Wallis test for numerical data, as appropriate. The significance level was set at p-value < 0.05. Patients’ EQ-5D-5L index was computed using disutility coefficients obtained from the Ethiopian general population [13].A multivariable Tobit regression model was employed to assess the association between EQ-5D-5L index, EQ-VAS, and potential predicting factors.

## Results

### Socio-demographic characteristics of study participants

The response rate was 100%. Of the study participants, 172 (50.7%) were female, and 167 (49.3%) were male. The majority (55.8%) of the study participants were less than 58 years old. Out of the study participants, 116 (34.2%) had had higher education. Moreover, 229 (67.6%) and 61 (18%) of subjects were married and widowed respectively (Table 1).

**Table 1:** Socio-demographic characteristics of the patients with hypertension in public hospitals, Addis Ababa, Ethiopia, 2021 (n = 339)

Variables		Frequency (%)
Sex		
Male		167 (49.3)
Female		172 (50.7)
Age (in years)		
< 58		189 (55.8)
≥ 58		150 (44.2)
Residence		
Urban		298 (87.9)
Rural		41 (12.1)
Marital status		
Single		23 (6.8)
Married		229 (67.6)
Divorced		26 (7.7)
Widowed		61 (18)
Educational status		
No education		43 (12.7)
Able to read and write		47 (13.9)
Primary		70 (20.6)
Secondary		63 (18.6)
Higher education		116 (34.2)
Occupation		
Employed		182 (53.7)
Non-employed		157 (46.3)
AMHI (ETB)		
< 1000		127 (37.5)
1000–4999		129 (38.1)
> 5000		83 (24.5)

*AMHI* Average monthly household income, *ETB* Ethiopian birr

### Clinical characteristics of hypertensive patients

Out of the study participants, 134 (39.5%) have had hypertension for more than 5 years. As for stage of hypertension, 135 (39.8%) had stage 1 hypertension and 90 (26.5%) had high-normal hypertension. Majority of the respondents, 267 (78.8%) were found to have comorbid illnesses (Table 2).

**Table 2:** Clinical characteristics of hypertensive patients attending public hospitals, Addis Ababa, Ethiopia, 2021 study participants (n = 339)

Variables	Frequency (%)
Duration of HTN	
< 1 year	26 (7.7)
1–5 years	179 (52.8)
> 5 years	134 (39.5)
Stage of HTN	
Normal BP	59 (17.4)
High-normal	90 (26.5)
Stage 1	135 (39.8)
Stage 2	48 (14.2)
Hypertensive crisis	7 (2.1)
Alcohol habit	
Yes	152 (44.8)
No	187 (55.2)
Smoking status	
Yes	40 (11.8)
No	299 (88.2)
Comorbidity	
Yes	267 (78.8)
No	72 (21.2)
Number of comorbidities	
One	121 (35.7)
Two	108 (31.9)
Three or more	38 (11.2)
Number of medications for HTN	
One	135 (39.8)
Two	159 (46.9)
Three or more	43 (12.7)
Number of medications (including HTN)	
One	54 (15.9)
Two	56 (16.5)
Three or more	227 (67.0)
History of hospitalization	
Yes	143 (42.2)
No	196 (57.8)
Past year hospitalization frequency	
Once	81 (23.9)
Twice	39 (11.5)
Thrice or more	23 (6.8)

*HTN* Hypertension

### Health-related quality of life among hypertensive patients

#### Health profile of hypertensive patients

**Fig. 1:**
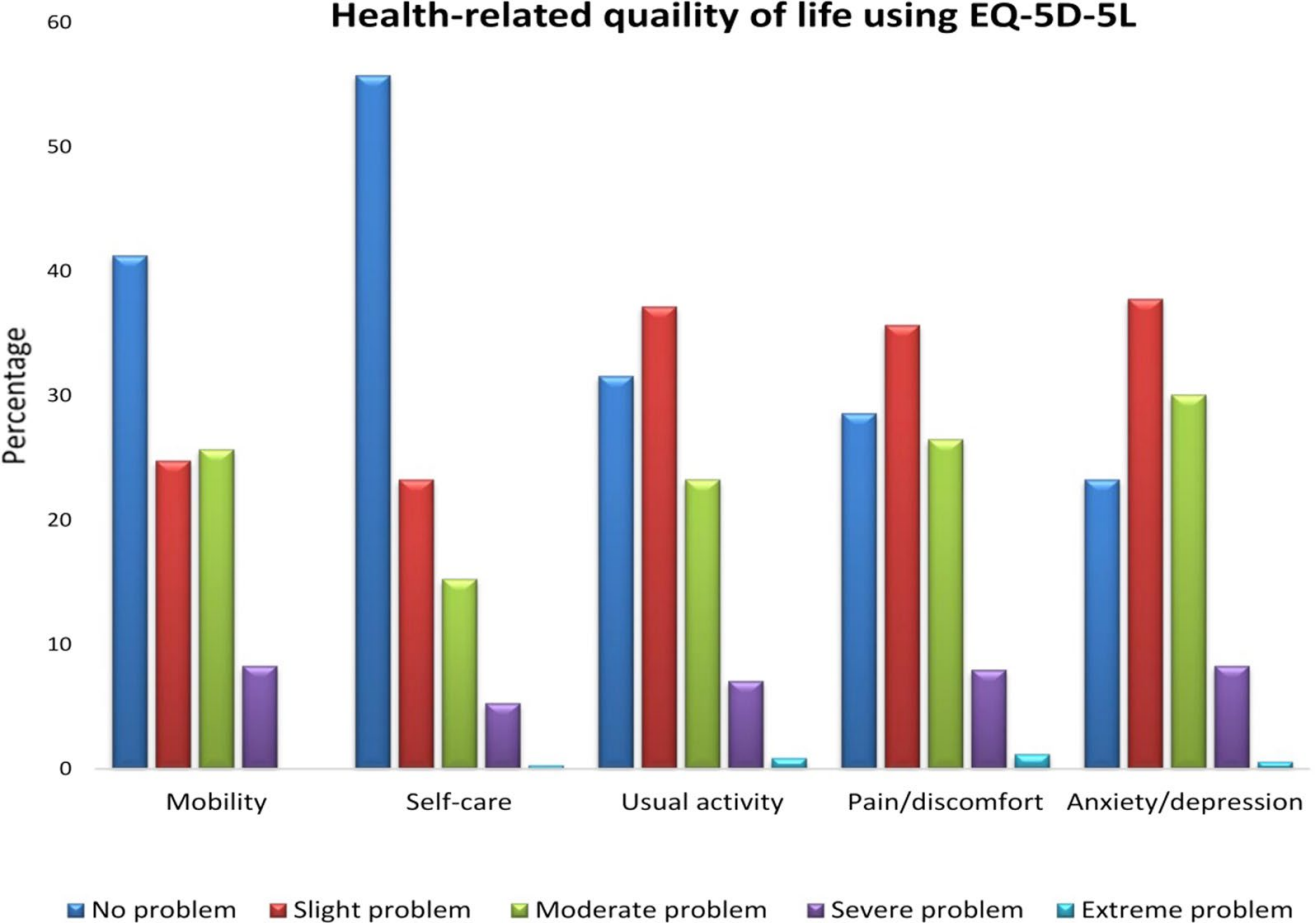
Health-related quality of life measured using EQ-5D-5L scale among hypertensive patients in public hospitals, Addis Ababa, Ethiopia, 2021 (n = 339)

Overall, 55.8 and 41.3% of the participants reported “no problem” in the self-care and mobility domains, respectively. In contrast, 37.2, 35.7, and 37.8% of participants reported at least slight problems with the usual activity, anxiety/depression, and pain/discomfort dimensions, respectively. The majority of the hypertensive patients (58.7%) had minor to severe problems in mobility, whereas 41.3% of them did not have difficulties in mobility, while 55.8% of the patients had no problem in self-care and 44.2% had minor to extreme problem in self-care (Fig. 1).

#### Socio-demographic, clinical characteristics, and reported health problems among participants

Concerning to participants’ reported problems based on their socio-demographic characteristics, 33.3, 26, 37.8, and 36.9% of female participants reported problems in mobility, self-care, usual activity and pain and discomfort dimensions, respectively (Table 3).

**Table 3:** Socio-demographic, clinical characteristics and percentage of the reported health problems among patients with hypertension in public hospitals, Addis Ababa

9Variables	% Reported problems				
	Mobility	Self-care	Usual activities	Pain/Discomfort	Anxiety/Depression
Total	58.7	44.2	68.4	71.4	76.7
Sex					
Male	25.7	18.2	30.7	34.5	37.8
Female	33.0	26	37.8	36.9	38.9
Age					
< 58	23.9	16.2	31.3	35.4	41.3
≥ 58	34.8	28.0	37.2	36	35.4
Residence					
Urban	49.9	36.9	59.9	61.4	66.4
Rural	8.8	7.4	8.6	10.0	10.3
Marital status					
Single	1.8	1.5	1.5	2.9	3.2
Married	36.6	24.2	44.2	45.1	50.4
Divorced	4.4	3.2	6.2	6.2	7.1
Widowed	15.9	15.3	16.2	17.1	15.9
Educational status					
No education	11.2	8.8	11.5	12.1	12.7
Read & write	9.4	8.6	10.9	10.6	12.1
Primary	16.2	12.7	16.8	17.7	14.7
Secondary	11.5	7.1	13.0	10.3	12.7
Higher education	10.3	7.1	16.2	20.6	24.5
Occupation					
Employed	20.9	14.5	29.2	33.6	38.9
Unemployed	37.8	29.8	39.2	37.8	37.8
AMHI					
< 1000	31.0	26.8	32.7	32.2	31.3
1000–4999	16.8	10.6	23.9	25.1	28.0
> 5000	10.9	6.8	11.8	14.2	17.4
Duration of HTN					
< 1 year	3.5	2.9	4.1	4.7	5.0
1–5 years	24.5	18.0	30.4	34.5	40.1
≥ 5 years	30.7	23.3	33.9	32.2	31.6
Stage of HTN					
Normal BP	7.1	3.8	9.4	12.1	12.1
High-normal	12.7	10.3	16.5	17.7	18.9
Stage 1	26.0	19.8	29.5	28.9	31.9
Stage 2	11.2	8.8	11.2	10.9	11.8
HYC	1.8	1.5	1.8	1.8	2.1
Alcohol habit					
Yes	23.0	17.1	27.7	32.2	36.0
No	35.7	27.1	40.7	39.2	40.7
Smoking status					
Yes	3.5	2.4	8.0	6.8	8.3
No	55.2	41.9	60.5	64.6	68.4
Comorbidity					
Yes	54.3	41.0	60.2	61.1	64.3
No	4.4	3.2	8.3	10.3	12.4
Number of comorbidities					
One	24.2	17.1	25.1	26.5	27.4
Two	20.4	15.9	24.5	24.5	27.1
≥ 3	9.7	8.0	10.6	10.0	9.7
Number of medications for HTN					
One	15.0	10.9	22.1	25.7	28.0
Two	33.0	26.0	33.3	34.8	36.9
> 3	10.6	7.4	12.4	10.3	11.2
Number of medications (including HTN)					
1	3.5	3.2	6.8	7.4	8.6
1	8.0	5.0	8.0	10.3	11.8
≥ 3	47.2	36.0	53.1	53.1	55.8
History of hospitalization					
Yes	31.9	26.0	37.2	37.5	37.2
No	26.8	18.3	31.3	33.9	39.5
Past year hospitalization frequency					
None	26.8	17.7	31.3	33.3	38.9
Once	15.9	12.7	19.8	20.9	20.1
Twice	9.4	8.3	10.9	10.6	10.9
≥ 3	6.5	5.6	6.5	6.5	6.8

#### Socio-demographic and clinical factors associated with health-related quality of life among hypertensive patients

Significant difference in EQ-5D-5L index values and EQ-VAS score was found among variables like marital status, educational level, household monthly income, duration of disease, stage of hypertension, total number of comorbid illnesses, number of anti-hypertension medications, total number of medications and past year hospitalization frequency at p-value < 0.05 by Kruskal–Wallis test. As well as age, gender, occupation, presence of comorbid illnesses, and hospitalization history were significant at p-value < 0.05 by the Mann–Whitney test (Table 4).

**Table 4:** Socio-demographic and clinical factors associated with EQ-5D-5L and EQ-VAS, among hypertensive patients in public hospitals, Addis Ababa, Ethiopia, 2021

Variables	EQ-5D-5L			EQ-VAS		
	Median (IQR)	Mean rank	P-value	Median (IQR)	Mean rank	P- value
Sex						
Male	0.89 (0.79–0.94)	185	0.07^a^	70 (55–85)	183	0.02^a^
Female	0.83 (0.72–0.92)	156		65 (50–79.8)	158	
< 58	0.91 (0.83–0.95)	205	0.00^a^	75 (60–85)	198	0.00^a^
≥ 58	0.77 (0.54–0.88)	126		60 (50–70)	135	
Residence						
Urban	0.87 (0.76–0.94)	177	0.001^a^	70 (55–80)	174	0.05
Rural	0.73 (0.47–0.89)	122		60 (50–70)	143	
Single	0.96 (0.92–1)	252	0.00^a^	80 (70–95)	241	0.00^a^
Marital status						
Married	0.88 (0.79–0.94)	185		70 (55–80)	178	
Divorced	0.85 (0.77–0.91)	158		66.5 (60–81.3)	186	
Widowed	0.73 (0.46–0.8)	89		55 (50–64.5)	105	
Educational status						
No education	0.73 (0.37–0.78)	87	0.00^a^	55 (50–65)	99	0.00^a^
Read and write	0.82 (0.54–0.94)	142		60 (50–75)	146	
Primary	0.80 (0.71–0.87)	133		60 (50–71.3)	134	
Secondary	0.87 (0.8–0.94)	190		70 (60–80)	185	
Higher education	0.92 (0.87–0.97)	224		80 (66.3–90)	220	
Occupation						
Employed	0.91 (0.85–0.97)	213	0.00^a^	75 (60–85)	208	0.00^a^
Unemployed	0.77 (0.52–0.87)	120		60 (50–70)	126	
AMHI						
< 1,000	0.74 (0.48–0.83)	108	0.00^a^	55 (50–65)	112	0.00^a^
1,000–4,999	0.91 (0.84–0.94)	204		75 (60–85)	204	
> 5,000	0.92 (0.84–0.97)	213		75 (60–87)	206	
< 1 year	0.89 (0.77–0.97)	189	0.00^a^	70 (50–90)	185	0.00^a^
1–5 years	0.91 (0.81–0.94)	197		70 (60–85)	193	
> 5 years	0.79 (0.57–0.87)	130		60 (50–72.8)	136	
Stage of HTN						
Normal BP	0.91 (0.83–0.95)	204	0.00^a^	70 (55–90)	198	0.00^a^
High-normal	0.91 (0.81–0.94)	195		75 (60–85)	200	
Stage 1	0.83 (0.73–0.93)	159		65 (55–75)	157	
Stage 2	0.79 (0.44–0.91)	127		57.5 (50–69)	122	
HYC	0.71 (0.23–0.76)	81		55 (50–80)	136	
Alcohol habit						
Yes	0.89 (0.76–0.93)	179	0.11	70 (51.3–80)	169	0.90
No	0.84 (0.73–0.93)	163		65 (55–80)	171	
Smoking status						
Yes	0.92 (0.84, 0.94)	210	0.06^a^	70 (60–80)	195	0.08
No	0.85 (0.74–0.94)	165		65 (52–80)	167	
Comorbidity						
Yes	0.83 (0.71–0.91)	149	0.00^a^	64 (50–75)	150	0.00^a^
No	0.94 (0.91–1)	248		82.5 (70–90)	243	
Number of comorbidities						
One	0.84 (0.74–0.93)	160	0.00^a^	68 (55–80)	169	0.00^a^
Two	0.84 (0.73–0.91)	153		60 (55–74.3)	148	
≥ 3	0.76 (0.37–0.83)	100		50.5(43.7–65)	97	
Number of medication						
One	0.91 (0.83–0.97)	211	0.00^a^	75 (65–85)	306	0.00^a^
Two	0.82 (0.71–0.92)	209		60 (50–75)	208	
≥ 3	0.77 (0.44–0.87)	152		57 (50–66)	150	
#of medication(including HTN)						
One	0.94 (0.89–1)	211	0.00^a^	80 (70–90)	306	0.00^a^
Two	0.91 (0.8–0.97)	252		75 (60–85)	241	
≥ 3	0.82 (0.71–0.91)	196		60 (50–75)	198	
History of hospitalization						
Yes	0.78 (0.54–0.87)	119	0.00^a^	58 (50–70)	128	0.00^a^
No	0.91 (0.82–0.97)	208		75 (60–85)	201	
Past year hospitalization frequency						
None	0.91 (0.82–0.97)	208	0.00^a^	75 (60–85)	202	0.00^a^
Once	0.83 (0.71–0.91)	137		60 (50–75)	146	
Twice	0.77 (0.54–0.87)	114		55 (45–70)	115	
≥ 3	0.53 (0.17–0.73)	55		51 (50–55)	77	

*AMHI* Average monthly household income, *ETB* Ethiopian birr, *HTN* Hypertension, *HYC* Hypertensive Crisis

^*a*^*P- value* < *0.05*

#### Factors associated with HRQoL among hypertensive patients

The EQ-5D-5L index values decreased with patients aged greater than 58 years. Patients with higher income levels greater than 5000 ETB had significantly higher index scores compared to less than 1000 ETB. Living in a rural area had a negative influence on HRQoL. In terms of clinical factors, the stage of hypertension was adversely associated with the index scores, where patients with a stage 2 hypertension had a lower index score than normal hypertension patients (Table 5).

**Table 5:** Factors associated with EQ-5D-5L and EQ-VAS score among hypertensive patients in public hospitals, Addis Ababa, Ethiopia, 2021

Variables	EQ-5D-5L			EQ-VAS		
	Coe	SE	p-value	Coe	SE	p-value
Sex (ref. = female)						
Male	0.014	0.023	0.532	1.446	1.632	0.376
Age category (ref. = < 58)						
≥ 58 years	− 0.072	0.027	0.08^a^	− 1.379	1.938	0.447
Residence (ref. = urban)						
Rural	− 0.139	0.034	0.00^a^	− 2.252	2.434	0.356
Marital status (ref. = single)						
Married	− 0.017	0.042	0.681	− 4.075	3.019	0.178
Divorced	− 0.037	0.055	0.503	− 1.869	3.979	0.639
Widowed	− 0.071	0.051	0.169	− 5.269	3.679	0.153
Educational status (ref. = no education)						
Able to read and write	0.026	0.039	0.501	5.665	2.819	0.045^a^
Primary	− 0.017	0.038	0.653	0.714	2.733	0.797
Secondary	0.020	0.044	0.648	4.639	3.193	0.147
Higher education	− 0.003	0.047	0.942	9.429	3.387	0.06^a^
Occupation (ref. = Employed)						
Unemployed	0.024	0.035	0.491	3.2664	2.534	0.199
AMHI (ref. = < 1,000)						
1000–4999	0.109	0.035	0.02^a^	6.683	2.498	0.08^a^
≥ 5,000	0.128	0.043	0.03^a^	7.308	3.067	0.02^a^
Duration of HTN (ref. = < 1 year)						
1–5	0.039	0.039	0.335	2.129	2.818	0.451
> 5 years	0.076	0.044	0.084	2.415	3.142	0.443
Stage of HTN (ref. = normal BP)						
High-normal	− 0.024	0.032	0.455	0.927	2.269	0.683
Stage 1	0.018	0.031	0.553	0.638	2.193	0.771
Stage 2	− 0.077	0.038	0.04^a^	− 2.812	2.759	0.309
Hypertensive crisis	− 0.105	0.075	0.161	1.021	5.377	0.849
Alcohol habit (ref. = yes)						
No	0.001	0.022	0.948	2.897	1.584	0.068
Smoking status (ref. = yes)						
No	− 0.033	0.033	0.320	− 1.302	2.373	0.584
Comorbidity (ref. = yes)						
No	0.070	0.039	0.074	7.241	2.808	0.010^a^
Number of comorbidities (ref. = one)						
Two	− 0.006	0.026	0.815	− 3.383	1.875	0.072
≥ 3	− 0.042	0.039	0.286	− 6.263	2.851	0.029^a^
Number of medications for HTN (ref. = one)						
Two	− 0.055	0.029	0.058	− 5.973	2.078	0.004^a^
≥ 3	− 0.101	0.039	0.012^a^	− 8.193	2.882	0.005^a^
Number of medications (including HTN) (ref. = one)						
Two	0.013	0.041	0.733	1.145	2.935	0.679
Three or more	0.020	0.051	0.694	3.084	3.688	0.404
History of hospitalization (ref. = yes)						
No	− 0.035	0.066	0.592	− 3.932	4.742	0.408
Past year hospitalization frequency (ref. = none)						
Once	− 1.128	0.067	0.057	− 9.459	4.822	0.051
Twice	− 0.122	0.071	0.085	− 11.234	5.111	0.029^a^
≥ 3	− 0.282	0.076	0.00^a^	− 10.349	5.483	0.060

*AMHI* Average monthly household income, *ETB* Ethiopian birr, *HTN* Hypertension

^*a*^*p-Value* < *0.05*

## Discussion

The study aimed to assess health-related quality of life and related factors in hypertensive patients at follow-up in public hospitals in Addis Ababa, Ethiopia. The mean total EQ-5D-5L Index score in the current study was 0.86. This is comparable to the study conducted in Palestine [15]. The index value recorded by patients or society varies from country to country and may be influenced by cultural beliefs [16].

The health index score is used to weight life years to calculate quality-adjusted life years, which is a summary measure of health gain that includes improvements in life expectancy and quality of life. It weighs the increase in life expectancy based on the quality of life experienced through the use of healthcare companies. As a result, an index value of 0.86 is assigned to a person with hypertension in a given health condition. Living in this state of health for ten years would be similar to living in perfect health for 8 years and 6 months [17]. This means that people with hypertension would rather live eight and a half years in good health than ten years in poor health. As a result, the index scores have been used to make health decisions that are important to patient health outcomes [18].

The median EQ-VAS score in this study was 69. The EQ-VAS scores were lower than the general population median of 90 [14], which may be acceptable since studies have shown that hypertensive patients have a lower quality of life than the general population [4, 19, 20]. Apart from the self-care dimension of the EQ-5D-5L, hypertensive patients had a higher incidence of health problems than the general Ethiopian population on the dimensions of mobility, habitual activities, pain and discomfort, anxiety, and depression [14]. It was found that the greatest agreement among hypertensive patients was questions on the anxiety/depression dimension, consistent with previous studies from other regions of the world [11, 21]. When evaluating the EQ-5D-5 results, the most common complaints were pain and discomfort. The self-care dimension appears to be least affected. This is consistent with the results of a previous study [22].

In addition, according to the descriptive profile of respondents, a higher proportion (41.3%) of the population aged 58 years reported having an anxiety/depression problem than older age groups. Therefore, to cope with anxiety and sadness, the younger population needs more psychological support and mental health interventions. The dimension of regular activities was problematic for more than half of the participants over 58 years of age. This may be related to age and comorbidities, underscoring the need for comorbidity management and palliative care for elderly hypertensive patients. Consistent with a recent systematic review and meta-analysis of observational studies of HRQoL in hypertensive patients, the results of the current study indicate that various socio-demographic and clinical factors are significantly associated with HRQoL in hypertensive patients [23]. Compared to the higher-income patient population, lower-income patients had a lower EQ-5D-5L index value and EQ-VAS score. Studies have shown that people with high blood pressure and other chronic conditions have a higher HRQoL when their financial situation improves [24, 25]. Improved health outcomes require health services and economic interventions, so social and financial support for low-income hypertensive patients should be addressed. In addition, the EQ-5D-5L index and the EQ-VAS score were essentially related to monthly household income and the number of antihypertensive drugs used. This agrees with the study conducted in Nepal [7].

According to our findings, the EQ-5D-5L index value decreased dramatically with age. HRQOL is most strongly influenced by age [26]. Biologically, aging is defined as the progressive and permanent accumulation of molecular and cellular damage, leading to loss of physiologic abilities and an overall deterioration of health [27, 28]. It was shown that the EQ-5D-5L index values of urban and rural hypertensive patients differ statistically. This is comparable to the study conducted in China [29]. This could be because the rural population had lower knowledge, attitudes and self-care practices regarding hypertension [30] and therefore negatively impacts HRQoL. Participants with a higher level of education reported higher EQ-VAS scores, while participants with a lower level of education reported lower EQ-VAS scores. This finding is consistent with research showing that more education improves quality of life [13, 31]. People with higher levels of education are more likely to be well-informed, have better critical thinking and decision-making skills, choose healthy lifestyles and preventive interventions, and use health-related knowledge to improve health outcomes and HRQoL [7]. A low EQ-VAS score was also associated with the presence of comorbidities. In addition to comorbidity, the number of comorbidities was significantly related to the EQ-VAS scores, similar to a study conducted in Canada [15]. As a result, counseling patients about lifestyle changes and medication adherence to prevent comorbidities, and promoting health education to reduce the extent of comorbidities can help improve their HRQoL.

## Limitations of the study

The study’s limitation is that generic instruments such as the EQ-5D-5L may not be sensitive enough or have insufficient symptom coverage to accurately quantify the impact of certain disorders on HRQoL.

## Conclusion and recommendations

The study results showed that high blood pressure had a negative impact on patients' well-being and HRQoL. Regardless of the HRQOL categories, a lower HRQoL level was associated with older age, patients living in rural areas, poorer educational attainment, lower monthly income, the presence and number of comorbidity, increased number of antihypertensive drugs, and frequency of hospitalization.

Emphasis should be given to patients with higher stages of hypertension, increased use of antihypertensive medications, and an increased hospitalization rate giving due focus to older, rural residents, and low-income patients to promote their health-related quality of life.

## Data Availability

The datasets generated and analyzed during the current study are available from the corresponding author on a reasonable request.

## Abbreviations

AMHI: Average Monthly Household Income
ETB: Ethiopian Birr
EQ-5D5L: Euro Quality of Life Group’s 5- Dimensions 5 Levels
EQ-VAS: Euro Quality of Life Group’s visual analog scale
EuroQol: European Quality of Life
HRQoL: Health-Related Quality of Life
IQR: Inter Quartile Range
NCDs: Non-Communicable Diseases
